# Delivery is key: lessons learnt from developing splice‐switching antisense therapies

**DOI:** 10.15252/emmm.201607199

**Published:** 2017-03-13

**Authors:** Caroline Godfrey, Lourdes R Desviat, Bård Smedsrød, France Piétri‐Rouxel, Michela A Denti, Petra Disterer, Stéphanie Lorain, Gisela Nogales‐Gadea, Valentina Sardone, Rayan Anwar, Samir EL Andaloussi, Taavi Lehto, Bernard Khoo, Camilla Brolin, Willeke MC van Roon‐Mom, Aurélie Goyenvalle, Annemieke Aartsma‐Rus, Virginia Arechavala‐Gomeza

**Affiliations:** ^1^ Department of Physiology, Anatomy and GeneticsUniversity of OxfordOxfordUK; ^2^ Centro de Biología Molecular Severo Ochoa UAM‐CSICCIBERERIdiPazUniversidad Autónoma de MadridMadridSpain; ^3^ Department of Medical BiologyUniversity of TromsøTromsøNorway; ^4^ UPMC, INSERM, UMRS 974CNRS FRE 3617Institut de MyologieParisFrance; ^5^ Centre for Integrative BiologyUniversity of TrentoTrentoItaly; ^6^ Centre for Amyloidosis and Acute Phase ProteinsDivision of MedicineUniversity College LondonLondonUK; ^7^ Grup d'Investigació en Malalties Neuromusculars i NeuropediatriquesInstitut d' Investigació en Ciències de la Salut Germans Trias i PujolBadalonaBarcelonaSpain; ^8^ Dubowitz Neuromuscular Centre and Developmental Neuroscience ProgrammeInstitute of Child HealthUniversity College LondonLondonUK; ^9^ Drug Discovery Informatics LabQasemi‐Research CenterAl‐Qasemi Academic CollegeBaka El‐GarbiahIsrael; ^10^ Drug Discovery and Development LaboratoryInstitute of Applied ResearchGalilee SocietyShefa‐AmrIsrael; ^11^ Department of Laboratory MedicineKarolinska InstituteStockholmSweden; ^12^ Institute of TechnologyUniversity of TartuTartuEstonia; ^13^ Centre for NeuroendocrinologyDivision of MedicineUniversity College LondonLondonUK; ^14^ Department of Cellular and Molecular MedicineUniversity of CopenhagenCopenhagenDenmark; ^15^ Department of Human GeneticsLeiden University Medical CenterLeidenThe Netherlands; ^16^ INSERM U1179UFR des sciences de la santéUniversité Versailles Saint QuentinMontigny‐le‐BretonneuxFrance; ^17^ Neuromuscular Disorders GroupBioCruces Health Research InstituteBarakaldoBizkaiaSpain

**Keywords:** antisense oligonucleotides, delivery, pre‐clinical models, RNA therapy, toxicity, Pharmacology & Drug Discovery

## Abstract

The use of splice‐switching antisense therapy is highly promising, with a wealth of pre‐clinical data and numerous clinical trials ongoing. Nevertheless, its potential to treat a variety of disorders has yet to be realized. The main obstacle impeding the clinical translation of this approach is the relatively poor delivery of antisense oligonucleotides to target tissues after systemic delivery. We are a group of researchers closely involved in the development of these therapies and would like to communicate our discussions concerning the validity of standard methodologies currently used in their pre‐clinical development, the gaps in current knowledge and the pertinent challenges facing the field. We therefore make recommendations in order to focus future research efforts and facilitate a wider application of therapeutic antisense oligonucleotides.

**Glossary**
Antisense oligonucleotides: Antisense oligonucleotides (AONs) are short strands of DNA or RNA that can bind to RNA through Watson–Crick base pairing and can modulate the function of the target RNA. Different types of AONs, defined by their chemical structure, are mentioned in this article: 2′ O‐methyl phosphorothioate oligonucleotides (2OMe), locked nucleic acids (LNAs), phosphorodiamidate morpholino oligomers (PMO) and peptide nucleic acids (PNAs). These “naked” antisense oligonucleotides can be combined with several moieties to increase their delivery, such as cell‐penetrating peptides (CPPs). These conjugated AONs include vivo‐morpholinos (VMO) or peptide phosphorodiamidate morpholino oligomers (PPMO). When AONs are used to disrupt RNA splicing, they are referred to as splice‐switching oligonucleotides (SSO), irrespective of their chemical structure.
Drug delivery systems: Drug delivery systems (DDS) are strategies to enhance delivery of drugs to target sites of pharmacological actions. Lipid nanoparticles (LNPs) or adeno‐associated virus (AAV) may be considered DDS.
Induced pluripotent stem cells: Induced pluripotent stem cells (IPSCs) are cells generated directly from adult cells, which may give rise to every other cell type in the body, and can propagate indefinitely.
Kupffer cells and liver sinusoidal endothelial cells: Both Kupffer cells (KCs) and liver sinusoidal endothelial cells (LSECs) constitute the hepatic sinusoidal lining, KCs are resident liver macrophages and form the greater part of the mononuclear phagocyte system, while LSECs are specialized endothelial cells with unsurpassed clathrin‐mediated endocytosis and endo‐lysosomal processing, enabling efficient scavenging of blood‐borne oligonucleotides, peptides, large macromolecules and nanoparticles. The space of Disse is the space between the liver sinusoidal lining and hepatocytes. Access to it is provided through fenestrae in LSECs or following transport through LSECs

## Introduction

**Figure 1:**
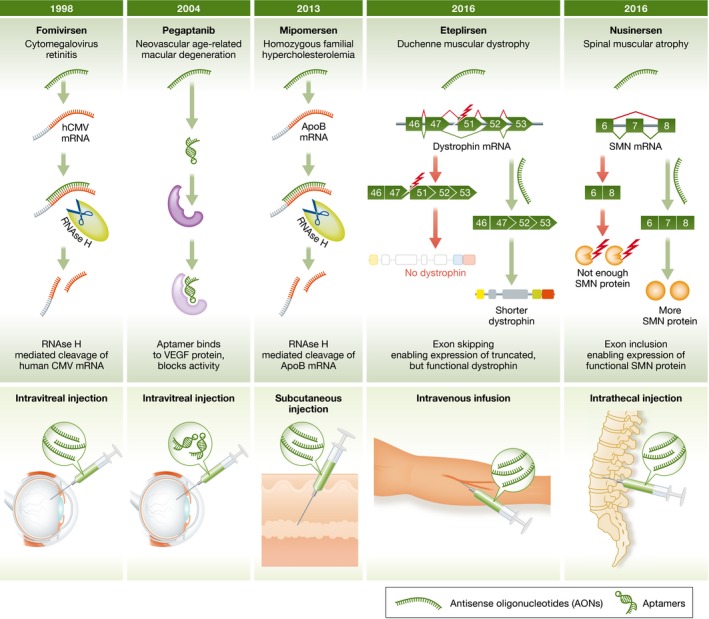
FDA‐approved antisense drugs^1^

Antisense oligonucleotides (AONs) are therapeutically attractive compounds; their mechanism of action is usually through hybridization to target sequences in pre‐mRNA or mRNA, and as such, AONs are highly specific. They can be manufactured at large scale in a standardized manner, and do not face many of the challenges of other genetic therapies such as gene addition and genome editing which need viral vector‐mediated delivery. Thus, it is not surprising that AON therapy development is a dynamic and active field. To date, four AON compounds have received marketing authorization and more than 100 clinical trials with antisense compounds are listed on ClinicalTrials.gov (Aartsma‐Rus, 2016; Fig 1).

**Figure 2:**
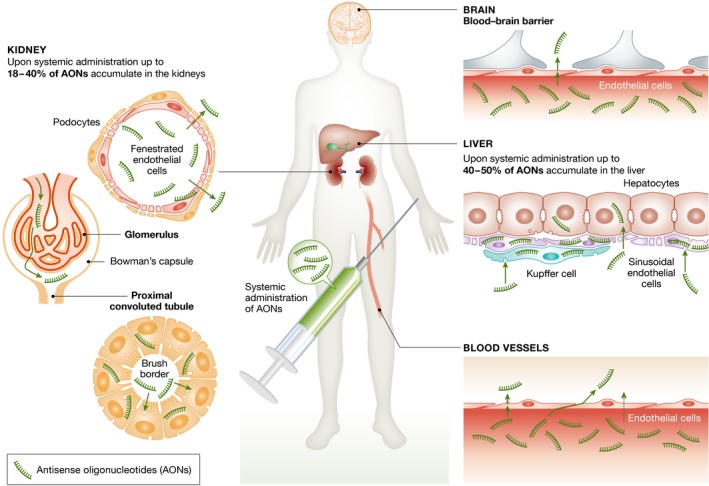
Barriers in AON delivery

One type of AON aims to modulate splicing; these so‐called splice‐switching oligonucleotides (SSOs) have been shown to restore protein expression in multiple clinical trials. However, following systemic administration the clinical benefit has been marginal and thus gaining regulatory approval has proved difficult. The ability of SSOs to induce sufficient levels of splice modulation in target tissues is limited by their poor delivery. Once in circulation, unmodified charged‐neutral AONs such as peptide nucleic acids (PNAs) and phosphorodiamidate morpholino oligomers (PMOs) are excreted rapidly via the kidney mainly as intact molecules typically with half‐lives of less than a few hours (McMahon *et al*, 2002; Amantana *et al*, 2007). It is assumed that on average < 1% of AONs reach the correct cellular compartment. Furthermore, due to the body's tissue barriers, the circulation of AONs is restricted, for example, most AONs are not able to reach the central nervous system (CNS) after systemic delivery (Fig 2). Significantly enhancing cell‐specific delivery of AONs is challenging due to our lack of knowledge of cellular uptake and subcellular mechanisms of transport and metabolism.

This manuscript focuses on AON delivery using systemic and localized administration, uptake mechanisms and model systems (Box 1). The aim of this publication is not to review what is currently known in the field of AON delivery; for this, we refer the reader to another recently published review (Juliano, 2016), rather we will use selected examples from both literature and experience to illustrate current challenges, problems and gaps in knowledge (Box 2). Using this approach, we outline what lessons can be learnt from previous work and suggest areas on which to focus future research efforts (Box 3).

**Box 1: Background to this manuscript**

- The delivery challenges facing AON therapy were recognized by the Cooperation of Science and Technology (COST) Action BM1207 (Networking towards clinical application of antisense‐mediated exon skipping for rare diseases [www.exonskipping.eu]).
- In order to address these challenges, four workshops were organized; the participants of which were both pre‐clinical and clinical researchers working on many aspects of AON therapy development.
- This manuscript is a result of discussions held at these meetings and focuses on AON delivery using systemic and localized administration, uptake mechanisms and model systems.

**Box 2: Key challenges of AON delivery**

- Target/off target effects.
- Toxicity due to AONs in entrapping tissues.
- Toxicity due to chemical modifications.
- Liver and kidney as a barrier.
- Tissue‐specific barriers (e.g. BBB for CNS).

**Box 3: Recommendations (or possible solutions?)**

- Make the most of “encapsulated tissues”.
- Develop efficient and safe drug delivery systems.
- Find specific receptor ligands.

## Delivery hurdles and how to make the most of them

### The problem of delivery

As with the development of any treatment, the therapeutic agent need only be effective in a subset of cells in the body. Most AON clinical trials have used the systemic intravenous administration route which results in the majority of AONs distributing to the liver, kidney, bone marrow, lymph nodes and a small part accumulating in adipocytes (Martin‐Armas *et al*, 2006; Geary *et al*, 2015). It is important to note however that as tissues consist of a mixture of cell types, not all cells within a tissue will take up equal amounts of AON. Increasing the administered dose in order to deliver sufficient amounts of AON to specific target cells is inherently limited by associated toxicities. Therefore, a detailed knowledge of what constitutes effective delivery within a specific disease context is essential in order to obtain sufficient potency with minimal toxicity.

**Figure 3:**
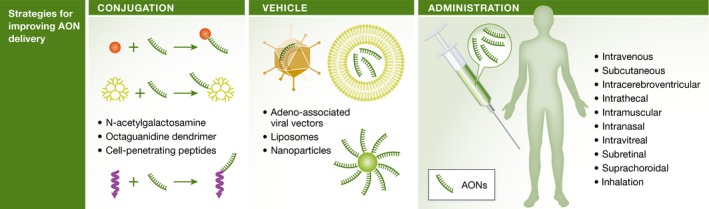
Strategies for improving delivery

Increases in the efficiency of AON delivery have been achieved through chemical modification, conjugation to other moieties as well as the development of new chemical backbones (Fig 3). While these modifications provide some benefits, questions surrounding pre‐clinical and clinical toxicity remain unresolved, and thus, it is important for scientists, toxicologists and pathologists as well as regulatory reviewers to be familiar with these issues.

### AON toxicity

The main toxicological challenges facing AON development programmes include: proinflammatory effects (vasculitis/inflammatory infiltrates), nephrotoxicity, hepatotoxicity and thrombocytopenia (Frazier, 2015). These types of toxicity are often called AON chemistry‐dependent toxicities, and represent effects that are not due to the Watson–Crick base pairing between an AON and an RNA sequence. These toxicities may still exhibit some sequence dependency despite the fact they do not involve base pairing. Such sequence‐specific toxicity has been observed with locked nucleic acids (LNAs) which, depending on their sequence, can cause profound hepatotoxicity as measured by serum transaminases as well as mild to severe liver lesions (Swayze *et al*, 2007; Stanton *et al*, 2012; Burdick *et al*, 2014; Kakiuchi‐Kiyota *et al*, 2014). This suggests that it may not be possible to define the toxicological profile of a new chemistry based on a limited number of sequences.

Hybridization‐independent toxicities fall into three general subcategories: AON accumulation effects, proinflammatory mechanisms (including immune complexes) and aptameric binding (as a consequence of AON interactions with extracellular, cell surface and/or intracellular proteins). The proinflammatory, aptameric binding effects are acute, while the accumulation effects are long term. As such, the relevance of the accumulation effect may depend on the type of treatment required by the pathology (high‐dose, short‐term treatment for cancer will not face the same cumulative effects as a lifelong therapy required for chronic diseases such as muscular dystrophies).

The mechanisms underlying these toxicities are also related to the specific chemical class of AON involved, and each class of agent has specific toxicity profiles. Phosphorothioate AONs have well‐characterized toxicities such as proinflammatory responses potentially related to their protein binding properties (Henry *et al*, 2002; Frazier, 2015), whereas neutral AONs such as PMOs do not interact to any significant extent with cellular proteins and tend to have fewer systemic toxicities.

Both the chemical backbone and specific sequence should therefore be taken into account when evaluating toxicological profiles of novel AONs. A number of reviews have now been published highlighting guidelines and summarizing consensus opinion on the appropriate strategies to use when assessing potential adverse AON‐mediated effects (Kornbrust *et al*, 2013; Engelhardt *et al*, 2015; Frazier, 2015).

### AON in the liver

With a blood flow of about 2 l/min and a sinusoidal blood lining surface area the size of a tennis court, the liver is one of the most vascularized tissues in the body. It is responsible for the clearance of large molecules and nanoparticles from blood, a function which is often counterproductive to the successful delivery of therapeutic compounds to other tissues or even specific cells within the liver. Several studies show that oligonucleotides (unmodified or conjugated) will end up in the liver to a far higher extent than the intended target tissue, although the rates vary between studies. In one study intravenous administration of an AON resulted in 40% and 18% accumulation in the liver and kidneys respectively (Bijsterbosch *et al*, 1997), whilst intravenous administration of CpG oligonucleotides resulted in 50% and 40% accumulation in the liver and kidneys respectively (Martin‐Armas *et al*, 2006). The main cellular site of liver uptake are the extremely active scavenger liver sinusoidal endothelial cells (LSECs) (Sorensen *et al*, 2015) followed by hepatocytes and Kupffer cells (KCs; (Bijsterbosch *et al*, 1997), however the degree of uptake in hepatocytes can vary from 40% in the first study to no apparent uptake in the CpG study. Similarly, a histological study revealed that phosphorothioate oligonucleotides accumulated mainly in KCs and LSECs (Butler *et al*, 1997). KCs specialize in phagocytic clearance of blood‐borne particles larger than 200 nm while LSECs mediate the clearance of smaller particles such as oligonucleotides, peptides, large macromolecules and nanoparticles via rapid and powerful clathrin‐mediated endocytosis (Sorensen *et al*, 2012). LSECs contain fenestrations with numerous open pores of 50–150 nm in diameter, enabling access into the underlying perisinusoidal space and therefore to hepatocytes. However, LSECs are also able to endocytose from the perisinusoidal space and do so at a much higher speed than hepatocytes (Magnusson & Berg, 1989). Stabilin is most likely the main receptor responsible for uptake of oligonucleotides in LSECs (Martin‐Armas *et al*, 2006). Our experience suggests that delivery reagents are necessary for the successful use of AON therapeutics in the liver, particularly targeting hepatocytes (e.g. for the treatment of hyperlipidaemia, hepatitis C or inherited metabolic disorders with major hepatic expression) (Disterer *et al*, 2013; Yilmaz‐Elis *et al*, 2013; Perez *et al*, 2014).

AON‐mediated liver toxicity, monitored as increased liver enzymes in the circulation, is generally considered a hepatocyte‐specific event (Kakiuchi‐Kiyota *et al*, 2014). However, it has been suggested that LSECs also play a significant role in the generation of liver toxicity caused by AONs. Firstly, as LSECs rapidly accumulate very high intracellular concentrations of AON due to the unsurpassed scavenger function of these cells, the adverse effects of oligonucleotides would be far more pronounced in these cells compared to other cell types. Secondly, it is known that initial damage to LSECs caused by certain drugs subsequently causes damage to the hepatocytes (DeLeve, 2007). It is therefore reasonable to assume that AON‐mediated liver toxicity is, at least in part, caused by initial damage to LSECs with subsequent injury to hepatocytes. Clearly, future attempts to unravel the mechanism of AON‐mediated hepatotoxicity must investigate LSECs in addition to hepatocytes and other types of liver cells.

### AON in the kidney

Renal blood flow through the glomerular capillary system efficiently clears a large portion of AONs from the bloodstream in a short time (up to 40% with some AONs). AONs appear to enter by receptor‐mediated endocytosis primarily at the brush border of the epithelium, although the specific receptor is as yet unknown. The fenestrated capillary endothelium provides a vast surface area for AON clearance, and in addition, AONs that are filtered through the glomerulus are reabsorbed by the proximal tubular epithelium via unidentified specific receptors, contributing to the high AON accumulation (Engelhardt, 2016). Following uptake, AONs are found in endosomes and lysosomes and high doses can result in the formation of cytoplasmic basophilic granules with or without vacuolation. The kidney accumulates one of the highest concentrations of AON following systemic administration in rodents, non‐human primates and humans, and this could make it the primary organ for toxicity. However, for 2′ O‐methyl phosphorothioate oligonucleotides, the histological changes seen in toxicity studies in animal models do not correlate with the data from multiple clinical trials that indicate no effect on renal function (Crooke *et al*, 2016; Engelhardt, 2016). Other chemistries may result in renal toxicity as has been described in clinical studies of LNA oligonucleotides (Engelhardt, 2016). Comparative pre‐clinical and clinical studies on renal toxicity are thus necessary for each AON chemistry, as well as basic research into delivery agents that target the specific cell type while minimizing renal clearance.

### Encapsulated tissues: a barrier has two sides

Recently renewed interest in the use of AONs to treat CNS diseases is based on the concept of the CNS as an encapsulated tissue; the same barriers that hamper delivery to the CNS after systemic delivery may trap therapeutic compounds once they reach the CNS. With standard systemic delivery, AONs have to cross the blood–brain barrier (BBB) or blood–cerebrospinal barrier, before they can distribute within the CNS. This barrier is comprised of a monolayer of endothelial cells, the basement membrane and either astrocytes or choroid cells which form tight junctions through interactions between these components (Palmer, 2010). Within the CNS, AONs benefit from a remarkably widespread distribution and exhibit efficient cellular uptake mechanisms (Whitesell *et al*, 1993; Rigo *et al*, 2014). The systemic route of delivery into the CNS includes diffusion (Banks *et al*, 2001) and receptor‐mediated endocytosis (Lee *et al*, 2002; Kozlu *et al*, 2014).

Direct delivery of AONs to the CNS is the most commonly used method of bypassing the BBB and can be achieved through intracerebroventricular or intrathecal (IT) injection. Due to the BBB preventing leakage of the AONs into peripheral circulation, relatively low doses can be administered less frequently (as half‐lives are increased), thus minimizing the risks of toxicity. To date, two phase I clinical trials have been completed using IT injection of AONs, one in amyotrophic lateral sclerosis (ALS; Miller *et al*, 2013) and one in spinal muscular atrophy (SMA) patients (Chiriboga *et al*, 2016) with encouraging results. Recently reported interim results from two phase III trials with nusinersen, the SMA therapeutic, were so positive that both trials were stopped early and all participants rolled over onto treatment immediately (Ionis Pharma press releases, currently accessible at http://ir.ionispharma.com/phoenix.zhtml?c=222170&p=irol-newsArticle&ID=2191319 and http://ir.ionispharma.com/phoenix.zhtml?c=222170&p=irol-newsArticle&ID=2220037). Nusinersen treatment was able to significantly improve achievement of motor milestones in infantile‐onset SMA (the most severe form of SMA) as well as in later‐onset (type II) SMA. The investigators were able to assess the uptake of nusinersen into the tissues of three infants that died during the trial and found that there was significant uptake of the AON into the CNS (including the target motor neurons, but also non‐neuronal cells) along with *SMN2* exon inclusion and expression of SMN protein (Finkel *et al*, 2016). It should be noted, however, that repeated IT therapy is a relatively expensive method of administration, necessitating specialist expertise and hospital visits.

A promising delivery approach is intranasal administration; molecules can be transported along the olfactory and trigeminal nerve pathways and the rostral migratory stream (Goyenvalle *et al*, 2015). Clinical trials utilizing this delivery route have resulted in improved cognition in Alzheimer's disease patients following application of intranasal insulin (Claxton *et al*, 2013). Among the CNS, the retina is becoming increasingly important as a target tissue for AON therapies (Bacchi *et al*, 2014). The eye is a small, enclosed, easy to access compartment and an immune‐privileged organ (Stein‐Streilein, 2008). Intravitreal, subretinal or suprachoroidal injections have been used (Thrimawithana *et al*, 2011). A well‐established example is the intravitreal treatment of cytomegalovirus‐associated retinitis in immunocompromised patients (Vitravene Study, 2002a,b,c), while topical and periocular routes are promising less invasive alternatives. Recently, a phase III study on a topical inhibitor of corneal angiogenesis (Cursiefen *et al*, 2009) significantly inhibited corneal neovascularization in patients with keratitis (Cursiefen *et al*, 2014). However, nucleic acids are retained by the superficial tissues and do not significantly penetrate intraocularly (Oliver, 1975; Bochot *et al*, 1998; Berdugo *et al*, 2003). Negatively charged AONs are potential candidates to be delivered into the eye by iontophoresis, which relies on applying a local electrical current (Andrieu‐Soler *et al*, 2006; Pescina *et al*, 2013).

It has been demonstrated that a number of modified AONs or those conjugated to different moieties (e.g. cell‐penetrating peptide [CPP]‐based delivery systems) (El‐Andaloussi *et al*, 2005; Du *et al*, 2011; Kang *et al*, 2014) can induce splice modulation in the CNS following systemic administration albeit at very low levels. To date, the most successful of these are tricyclo‐DNA (tcDNA) oligonucleotides (Goyenvalle *et al*, 2015).

### AAV vectors as an alternative delivery strategy for antisense sequences

An alternative way of antisense sequence delivery is the use of adeno‐associated virus (AAV) vectors expressing the antisense sequence. AAVs are promising vectors for *in vivo* therapeutic gene delivery and have been shown in a number of human clinical trials to deliver therapeutic genes to a variety of organs and tissues including the CNS, liver and muscle (Mingozzi & High, 2011). The first AAV‐based gene therapy was approved in the European Union in November 2012 for the treatment of lipoprotein lipase deficiency (Glybera; Kastelein *et al*, 2013). While AAV vectors can be used for gene transfer, they also offer an alternative strategy for antisense sequence delivery. A one‐shot injection of AAV vectors expressing the antisense sequence disguised in U7 snRNA (AAV‐U7) or U1 snRNA (AAV‐U1) induces splice modulation in skeletal muscles as demonstrated in pre‐clinical work for Duchenne muscular dystrophy (DMD) (Goyenvalle *et al*, 2004; Denti *et al*, 2006a,b, 2008; Vulin *et al*, 2012; Le Guiner *et al*, 2014). The main limitation to this approach is that the immune response prevents repeated AAV treatment (Lorain *et al*, 2008). It is therefore necessary to efficiently induce a lasting therapeutic benefit via a single dose. Furthermore, as for AON delivery, the pathology could affect AAV therapeutic efficiency, as is the case for AAV‐U7 in dystrophic DMD muscles: the altered membranes of dystrophic myofibres are an advantage for AON uptake but a limitation for AAV genome maintenance (Vulin *et al*, 2012; Le Hir *et al*, 2013) although introducing a pre‐treatment may help to negate this effect (Peccate *et al*, 2016).

In order to develop AAV‐delivered antisense RNA further, issues with large‐scale production to good medical practice (GMP) standards, immune response to the vector and persistence of the viral genomes in target tissues will need to be addressed.

### Recommendations

The majority of pre‐clinical studies focus on the target tissue to assess AON efficacy, yet there needs to be an early emphasis on assessing uptake in tissues such as liver and kidney due to their influence on systemic delivery. This is particularly important when developing new generations of AONs or different drug delivery systems (DDSs) which might increase the uptake in the targeted tissue but also in unintended tissues. The ratio of targeted versus unintended uptake should therefore always be considered. Similarly, in the context of improving AON delivery, evaluation of toxicity is often neglected as efficacy is generally the primary objective. It is also important to note that toxicity thresholds vary between species as demonstrated by a peptide‐conjugated PMO (PPMO) targeting the human dystrophin exon 50 (AVI‐5038), which following pre‐clinical work, was found to cause tubular degeneration in the kidneys of cynomolgus monkeys (Moulton & Moulton, 2010). However, numerous specific and early biomarkers of toxicity can now be evaluated in mice (treated with higher doses of AONs) or rats to predict toxicity in pre‐clinical development.

Through the discussion of confidential data during our action workshops, it became clear that these unintended uptake and toxicological challenges should be addressed in the very early stages of new AON development. It was also discussed that negative data such as chemical modifications that reduce delivery as well as those that increase toxicity are rarely published. A relevant example is the data on the inherent toxicity of vivo‐morpholinos (VMOs). VMOs are morpholino AONs covalently linked to an octaguanidine dendrimer to improve *in vivo* delivery. Initially, no toxicity data were available, but following networking events, we learned that several groups had observed lethargic behaviour in mice immediately after intravenous injection (10–50 mg/kg), with mortality as high as 20% within 12 h. These observations, as well as an article describing an alteration in the clotting system inducing cardiac arrest as the possible cause of death, were later published (Ferguson *et al*, 2014; Gallego‐Villar *et al*, 2014). The observed toxicity clearly limits the potential clinical application of VMOs and underscores the importance of making the scientific community aware of negative results as early as possible.

## AON uptake mechanisms

### The cellular uptake journey

While our understanding of the cellular transport machinery and trafficking system in general is expanding, knowledge is still relatively sparse in terms of its regulation and in particular regarding how to take advantage of these complex events from a delivery perspective. The uptake journey can be divided into four stages: (i) endocytosis via phagocytosis, macropinocytosis, micropinocytosis via clathrin and caveolin‐independent pathways, caveolar internalization and classical clathrin‐mediated endocytosis; (ii) intracellular trafficking of endosomes regulated by Rab, SNAREs and tethering proteins; (iii) escape from the endosomal compartment, thought to occur especially during the membrane fusion events of intracellular trafficking; and (iv) nuclear entry, both actively mediated by nuclear pore mechanisms and passively via simple diffusion (Juliano *et al*, 2012).

In order to improve the delivery and bioavailability of therapeutic AONs, such as SSOs, a variety of different DDSs have been utilized (Juliano, 2016). The main strategies include either making modifications directly on the AONs (conjugation of targeting ligands or delivery components) or incorporating AONs within the DDSs (various nanoparticle‐based approaches). Taken into account our present understanding of the general delivery process, we can, to an extent, chemically programme the DDSs in an attempt to surmount and control the barriers for efficient and selective uptake (Fig 3). This question has been studied most extensively in two non‐viral vector classes, lipid nanoparticles (LNPs) and CPPs.

With regard to the endocytotic and intracellular trafficking steps, it has also been demonstrated that exocytosis and re‐uptake can play an important role in uptake and trafficking of LNPs with nucleic acids (Sahay *et al*, 2013). Strategies to prevent the exocytosis of internalized AONs, for example by inhibiting the activity of the cholesterol efflux regulator Niemann–Pick‐like C1 (NPC1), may therefore be employed in future designs to improve efficiency of AON endocytosis.

After entering cells via endocytosis, DDS plus AONs move to, and to a greater extent get entrapped in, endo‐ or lysosomal compartments. This so‐called endosomal entrapment is also considered the main rate‐limiting step in DDS/AON delivery and has long since been regarded as the main hurdle to overcome to improve nucleic acid delivery (El‐Sayed *et al*, 2009). Despite extensive efforts, only a relatively small proportion of the DDSs/AONs escape from endosomes (Gilleron *et al*, 2013). Consequently, a variety of modifications have been designed that are aimed at increasing the endosomal escape of DDSs/AONs. The main line of development is based on the compounds that are protonated in the low‐pH endosomal compartment and provide escape through the so‐called proton sponge effect, for example different ionizable lipids, polyamidoamine‐based polymers or modified peptides. Another approach has been to utilize different hydrophobic modifications, which enhance the degree of membrane affinity of the DDSs.

CPPs have been used both as direct chemical conjugates with charge neutral AONs (such as PMO or PNA) and as nanoparticle‐based formulations (Boisguerin *et al*, 2015; Lehto *et al*, 2016). Although CPPs were thought originally to directly translocate across membranes, more recent work suggests that the positively charged CPPs interact with negatively charged cell surface proteoglycans before internalization by a variety of endocytic pathways. In the context of SSO delivery, covalent conjugates of CPPs to PMOs (peptide‐PMOs, PPMOs), have received considerable attention and have been used successfully in pre‐clinical models of DMD (Yin *et al,* 2010; Godfrey *et al,* 2015). PPMOs with designed amphipathicity have been used to great effect in providing enhanced affinity towards membranes, including the endosomal membranes, and significantly enhanced the endosomal escape capacity and delivery efficacy of the AONs. Similarly to LNPs, it has recently been demonstrated with CPPs used for SSO delivery that their association and uptake is to a considerable degree mediated by scavenger receptors (especially class A) (Ezzat *et al*, 2011). Preliminary data suggest that the activity of certain CPPs is also dependent on the degree of re‐export following endocytosis, similar to the observations for LNPs (unpublished data).

### A note on the concept of targeted delivery

As mentioned previously, it is imperative for any DDS/AON to be as specific to the target tissue as possible, and the so‐called targeted delivery approaches have been under investigation. Truly targeted delivery would ideally facilitate increased delivery to the nucleus in a subset of cells in a specific part of the body, while selectively reducing delivery in non‐diseased tissues and especially in those in which toxic side effects manifest, for example, by taking advantage of specific receptors and/or using shielding strategies. Novel methods to increase delivery are generally referred to as “targeted delivery” platforms, yet this term often refers to improvements seen in delivery to a specific tissue target but does not rule out increased oligonucleotide delivery to other tissues (Yin *et al*, 2009).

Different receptor ligands have been conjugated directly either to the AONs or to the DDS systems to enhance the affinity towards specific tissues usually overexpressing these receptors. A recent example is the utilization of N‐acetylgalactosamine (Gal‐NAc) conjugates that mediate endocytosis via the asialoglycoprotein receptor (Akinc *et al*, 2010; Rajeev *et al*, 2015), thereby increasing both uptake efficiency and providing hepatocyte‐specific targeting. Notably, lipid‐based delivery systems primarily deliver to the liver where they display very high activity. Recent studies with LNPs have demonstrated that the likely reason behind this strong tropism towards liver is that they bind to ApoE in circulation and are efficiently taken up by hepatocyte LDL receptors and scavenger receptor‐BI (Akinc *et al*, 2010).

A proper assessment of global biodistribution and delivery following systemic administration in disease models at present requires cumbersome whole‐animal studies and studies of this type are scarce in the literature. However, where performed, these whole‐animal studies show that relatively unmodified AONs do distribute to a wide variety of organs and tissues (Geary *et al*, 2015). Even if biodistribution studies show that AONs are distributed to a particular organ, this may not necessarily mean that the AONs are reaching the particular type of cell/tissue within the organ in question, for example hepatocytes as opposed to LSECs and KCs within the liver.

### Recommendations

More focus is needed on evaluating the biodistribution of AONs at the organ, tissue and cell level. Measuring the presence of AON commonly relies on hybridization assays and/or mass spectrometry and although some provide *in situ* detection methods (Goebl *et al*, 2007), most use whole tissue lysate preparations (Yu *et al*, 2002; Heemskerk *et al*, 2010; Verhaart *et al*, 2014; Burki *et al*, 2015; Goyenvalle *et al*, 2015). The development of methods that provide information on subcellular localization for all AONs would be advisable.

Improvements in AON delivery can be driven by two main lines of development: the evolution of AON chemistry itself and/or the utilization of efficient DDSs. The successful application of these may depend on the disease context and specific tissues in question. The development of tcDNA demonstrates how a change in chemistry can improve both the uptake and trafficking profile of an AON (Renneberg & Leumann, 2002; Goyenvalle *et al*, 2015). For clinical translation of many AON chemistries, it may be envisioned that some form of drug delivery component will be necessary. Nevertheless, in either scenario, to be able to rationally programme the delivery, including cellular association, uptake and trafficking of AONs, further basic research is required to fully characterize and potentially exploit these complex events.

## Model systems used for pre‐clinical development of AONs

**Figure 4:**
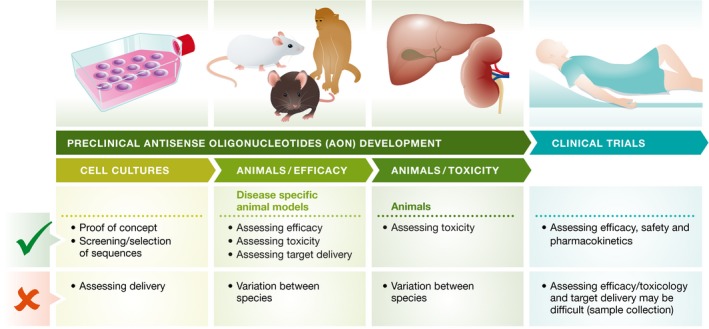
Stages in pre‐clinical AON development

Both *in vitro* and *in vivo* models are required for pre‐clinical testing of new AONs. It is generally accepted that while *in vitro* models provide data on the AON mechanism of action and efficacy, *in vivo* models are better suited to assess the delivery of the compound. Therefore, most AON sequence variants are pre‐screened *in vitro* and only candidates deemed promising are then progressed to *in vivo* screening (Fig 4).

### Cell culture models

As is the case of SSOs currently under investigation for the treatment of DMD, the most efficient AON sequence of a given chemical backbone can be identified following AON treatments performed on cell lines derived from DMD patients (Aartsma‐Rus *et al*, 2003; Arechavala‐Gomeza *et al*, 2007). It is important to use an appropriate cell line, due to cell‐specific repertoires of splicing proteins which may confer different rankings of effective AONs (Garanto *et al*, 2015). Many cell lines derived from animals and humans have been used to select antisense sequences targeting different disorders and AONs are usually transfected into cells using chemical agents or by electroporation. Recent studies showed that certain cells can take up AONs directly from the medium with no transfection agent required: this process is called “gymnosis” and requires longer incubation times (3–10 days) to generate RNA inhibition effect (Stein *et al*, 2010). It is thought that uptake of AONs with no chemical/physical modification is possibly due to an endocytosis process, and some groups have attempted to identify endogenous AON receptors. No definitive results have been provided, although a list of putative candidates has been suggested (Juliano *et al*, 2012).

Patient cell lines derived from available tissue (e.g. skin fibroblasts or lymphoblasts) do not always recapitulate tissue‐specific disease settings, and therefore, patient‐specific induced pluripotent stem cells (iPSCs) provide a compelling alternative allowing differentiation towards relevant cell types to model disease. In Leber congenital amaurosis, a retinal dystrophy causing childhood blindness, iPSCs derived three‐dimensional optic cups and retinal epithelium have been used to test therapeutic AONs (Parfitt *et al*, 2016) while SMA patient‐derived iPSC‐differentiated motor neurons have also been used with success to test AON therapy (Nizzardo *et al*, 2015).

Although cell cultures are mostly used to test the efficacy of the AONs, these same cultures are often used to test AON delivery potency and half‐life after administration. Carcinoma cells (HeLa, MCF‐7, MDA‐MB‐231, A375) have been used to test CPP antisense efficacy as well as other AON delivery methods (antisense antibody conjugates with lysosome or RGD‐conjugated antisense) (Vasconcelos *et al*, 2014) and suspension cell lines derived from peripheral blood cells of leukaemia patients (KASUMI, MV4‐11, K562 and AML) were used to test the uptake of cenersen, an antisense drug developed to shut down toxic chemotherapeutic effects (Alachkar *et al*, 2012).

### Animal models

Toxicity studies are generally performed in wild‐type rats and non‐human primates. However, *in vivo* proof‐of‐concept studies are mostly done in mouse disease models, because this allows assessment of target mRNA and protein as well as on a functional (or therapeutic) level. An additional advantage of using disease models derives from the fact that sometimes the pathology affects delivery. This can have positive consequences, for example it has been shown that the altered BBB in some CNS diseases favours AAV entry (Chen *et al*, 2009) and a more permeable endothelium in the dystrophic muscle of the *mdx* mouse model for DMD facilitates AON uptake (Heemskerk *et al*, 2010). The opposite could also be envisaged: for example, limited uptake in dystrophic muscle may occur due to increased fibrosis, therefore impeding delivery.

*In vivo* bio‐imaging (*in vivo* time‐domain optical imaging) could represent a relatively inexpensive, robust and fast way to obtain information about biodistribution and body “half‐life”/clearance of fluorophore‐labelled AONs in nude mice. Only minute amounts of compound are needed (10 μg per mouse), compared to mg scale for *in vivo* efficacy studies. Tissue accumulation can also be determined by subsequent *ex vivo* scans of excised tissues/organs. This method can be exploited for the initial screening of antisense agents and their conjugates *in vivo* and used to evaluate candidates to be selected for AON efficacy studies in mouse disease models. However, although labelled AONs can provide useful information on cellular uptake and biodistribution in both cell and animal models, these experiments are limited by the unknown ways in which these processes are affected by the tag itself as well as the stability of the labelled AON or AON/DDS (Falzarano *et al*, 2014; Lehto *et al*, 2014).

### Recommendations

*In vitro* and *in vivo* models are complementary and their use will have to be preceded by an understanding of the limitations of each model. Cell culture models are useful for proof‐of‐concept studies as long as an adequate cell type is selected, not only considering their target pre‐mRNA, but also the spliceosome expressed in different cell types. The possibility of deriving diverse cell types from affected patient's iPSCs offers an interesting alternative for testing AONs in different cellular environments, however, to date this approach is still uncommon. Delivery studies in cell culture provide extremely useful data about cellular uptake mechanisms, target receptors and antisense metabolism, but these results cannot be extrapolated to, nor substitute for, *in vivo* studies testing the delivery capabilities of different AONs or AONs/DDS combinations.

The progression of disease in animal models needs to be well characterized as the timing of intervention needs to be therapeutically appropriate. One may be inclined to use animal models to assess whether treatment can prevent pathology; however, in patients there is often a certain amount of pathology present when the disease is diagnosed, so evaluating the therapy after onset of pathology may provide more realistic information on the therapeutic effects. The use of a humanized mouse model allows the evaluation of the exact AON to be used in clinical trial, rather than using mouse‐specific counterparts. These models are not always available, but with the appearance of CRISPR/Cas9 technology, generating them could now be relatively straightforward. However, inserting the human gene with the human mutation does not suffice to completely humanize the system, as the splice‐regulating proteins are those of the mouse (Garanto *et al*, 2015, 2016). Alternatively, non‐human primates might be envisaged as a suitable species for testing of the effects of AONs designed for the treatment of human disease, as their genomic sequences are closer to those of humans, as is their metabolism, but this needs to be balanced against the ethical issues of using such animals and the increased expenses incurred.

Both cell culture and animal models are therefore complementary and indispensable for the development of AON‐based therapies and they should be used with foreknowledge of their limitations.

## Concluding remarks

Antisense oligonucleotides as RNA‐modulating therapeutics are highly specific and easy to design making them attractive sequence‐specific drugs and their role in the pipeline towards “personalized therapy” has made them a hot topic of research in recent years. The poster boy for this development has been DMD and stakeholders representing other rare disorders have followed the advances in the development of these AONs closely. However, despite several companies involved and many compounds in the pipeline, results from the first clinical trials have been disappointing (Goemans *et al*, 2011; Mendell *et al*, 2013), particularly when compared with pre‐clinical *in vitro* and *in vivo* studies of these molecules. Although there are many aspects to improve upon in the planning and evaluation of many of these compounds (Straub *et al*, 2016), all of the AONs tested in DMD have shown low effectiveness and this has been linked to their deficient delivery.

Compounds currently in the clinic are referred to as “first generation” and with all subsequent generations referring to compounds that aim to improve delivery of these drugs by different means. However, there are big gaps in our current knowledge of the delivery process of these molecules and these gaps can only be filled by basic research, collaboration and publication of negative results. Regrettably, basic research is badly underfunded in favour of highly translational projects with proven impact.

There are some positive steps being taken in the right direction; networks such as the EU's COST Actions have fueled collaboration and data sharing and initiatives have encouraged the publication of negative data, yet more effort is needed to direct funding towards basic research questions.

DMD has been leading the race for some time, however, it is unlikely to be one of the first disorders to benefit fully from AON therapy, as there are still many issues that need to be addressed. The decision of one of the main companies developing AONs to halt the clinical development of all its first‐generation AONs could slow the pace of the whole field (https://www.wsj.com/articles/biomarin-to-stop-developing-current-drugs-for-duchenne-muscular-dystrophy-1464733329). On the other hand, FDA's accelerated approval of eteplirsen despite the small sample size of their main clinical trial (FDA press announcement, currently accessible at http://www.fda.gov/NewsEvents/Newsroom/PressAnnouncements/ucm521263.htm) may have the opposing effect. Despite drug approval, it is not guaranteed that AONs will deliver on their promise. Eteplirsen costs an estimated $400,000 per patient per year and US insurance companies seem reluctant to reimburse the drug, arguing that no functional effect has yet been shown (Aartsma‐Rus & Krieg, 2017).

Regardless of what the future holds for DMD AONs, the combined knowledge accumulated during their research will make it feasible for other disorders, particularly those that target more accessible compartments, to benefit from the clinical use of this technology soon, as was recently seen with the encouraging results of the phase II and phase III nusinersen studies (Finkel *et al*, 2016) (Ionis Pharma press releases, currently accessible at http://ir.ionispharma.com/phoenix.zhtml?c=222170&p=irol-newsArticle&ID=2191319) and its accelerated approval by the FDA [FDA press release on nusinersen approval (2016), currently accessible at www.fda.gov/NewsEvents/Newsroom/PressAnnouncements/ucm534611.htm]. In the case of DMD, eteplirsen and other first‐generation AONs are likely only to provide limited benefits to patients until the next‐generation compounds arrive. For that to happen and for other AONs to reach the clinic, research into the delivery issues mentioned in this article is vital (Box 4).

**Box 4: Pending issues**

**Delivery should be prioritized from the outset of drug development**

Delivery optimization is often left till the latter stages of pre‐clinical development (often following rigorous AON sequence optimization) yet it is crucial to address this issue earlier as it has proven critical to the successful translation of AON therapies.

**Further basic research is needed to fully characterize the delivery process of AONs**

Gaps in our knowledge of the mechanisms governing the delivery of these molecules need to be filled in order to rationally programme the delivery, including cellular association, uptake and trafficking of AONs.

## Conflict of interest

B.S. is the founder and CEO of D'Liver, NO. V.S. is supported by funding provided by Sarepta Therapeutics, US. W.v.R.M. is the main inventor on two published patent applications WO2012/018257 and WO2015/053624 regarding exon skipping approaches for neurodegenerative diseases and co‐inventor on one provisional patent US 62/315,512. A.A.R. reports being employed by LUMC, which has patents on exon skipping technology. As co‐inventor on some of these patents she stands to gain financially from potential royalties and she is also on the SAB of Philae Pharmaceuticals and ProQR and has been *ad hoc* consultant for Summit PLC, BioClinica, GLG consulting, Deerfield, Global Guidepoint, PTC Therapeutics, BioMarin and BMS and has presented at satellite symposia organized by PTC and BioMarin. Remuneration for SAB and consulting activities and speaker honoraria went to her employer. All other authors declare no conflicts of interest.

## Footnotes

1 Correction added on 2 May 2017 after first online publication: text stating nusinersen is seeking approval was removed from the Fig 1 title, as it is already approved.
